# Of starch and spit

**DOI:** 10.7554/eLife.47523

**Published:** 2019-05-14

**Authors:** Mareike C Janiak

**Affiliations:** Department of Anthropology and ArchaeologyUniversity of CalgaryCalgaryCanada

**Keywords:** commensalism, adaptation, evolution, amylase, starch, saliva, Human, Mouse, Rat, Rhesus macaque, Other

## Abstract

Animals living alongside humans have multiple copies of the gene for alpha-amylase, the enzyme that breaks down starchy foods, and high levels of this protein in their saliva.

**Related research article** Pajic P, Pavlidis P, Dean K, Neznanova L, Romano R, Garneau D, Daugherity E, Globig A, Ruhl S, Gokcumen O. 2019. Independent amylase gene copy number bursts correlate with dietary preferences in mammals. *eLife* **8**:e44628. doi: 10.7554/eLife.44628

Rice, bread and potatoes are just a few of the starchy foods that many human populations rely on in their day-to-day lives. In fact, starches have probably been part of our diet for a long time. Starch residues have been found in cooking hearths from 120,000 years ago, when the Sahara desert was still covered in lush vegetation, and cereals were some of the first crops to be domesticated and farmed (Larbey et al., 2019; Simmons, 2011). This close relationship with starch has inevitably left its mark on our genes.

Starch is formed of many sugar molecules attached together, so it must be cut into smaller pieces to be absorbed into our bloodstream: the cutting up process is guided by an enzyme called alpha-amylase. This protein is encoded by the *AMY* gene family, and in a few species such as humans, it is expressed in both the pancreas and saliva.

Modern humans differ from other primates, and even other species of early humans, in how many copies of the *AMY* genes we have. Unlike Neanderthals and Denisovans, who had only two diploid copies, we carry up to 20 copies of the *AMY1* gene, which produces salivary amylase. In fact, there is evidence that this gene was strongly selected for as early as the middle Pleistocene, when modern humans emerged (Inchley et al., 2016). This may suggest that our lineage has evolved specific adaptations to digest starch-rich foods, underlining the long and continuing importance of these staples in our diet (Hardy et al., 2015).

Several studies have found that the higher the number of *AMY1* gene copies, the more amylase protein is expressed in saliva (Perry et al., 2007; Carpenter et al., 2017). This may affect how well we digest starchy foods, but also how we may perceive them. Indeed, as the amylase in saliva breaks down starch in the mouth, it releases a sweet taste that we can detect (Mandel et al., 2010; Mandel and Breslin, 2012).

Of course, humans vary in how much starch they consume; strikingly, populations that have higher numbers of amylase gene copies and more amylase in their saliva also tend to eat more starch (Perry et al., 2007; Inchley et al., 2016). A similar pattern is found in wolves and their domesticated counterpart, the dog. While wolves only have two diploid copies of the amylase gene, dogs can have many, and they also have higher levels of amylase expression and activity in their pancreas (Axelsson et al., 2013). As wolves became dogs, their behavior and appearance changed, but they also adapted to a new source of food that was rich in starch: human food scraps (Figure 1A,B).

**Figure 1.**
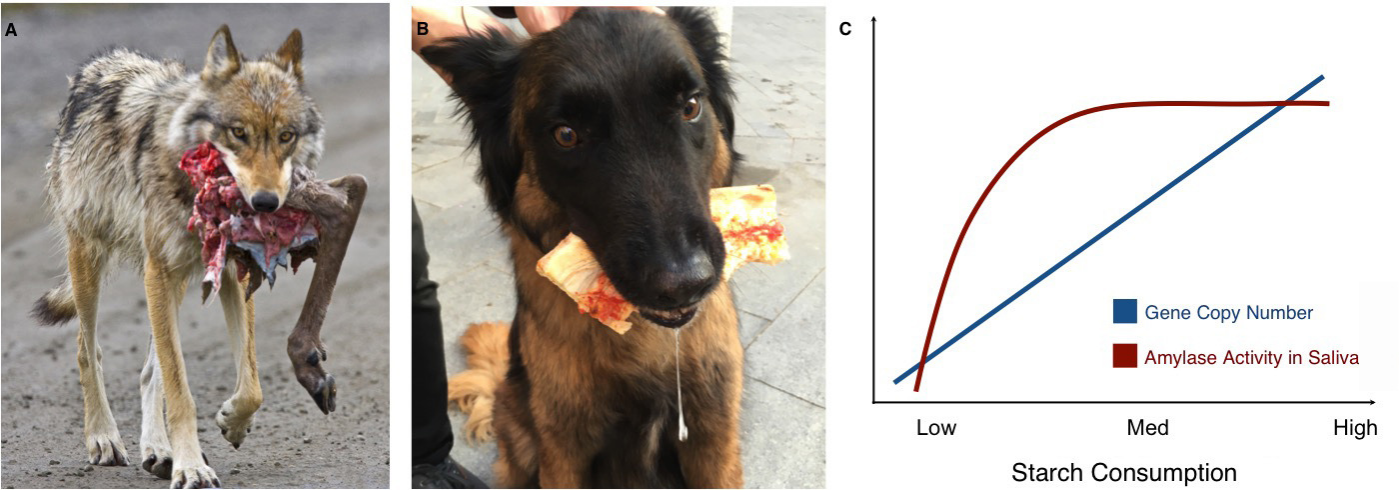
Links between proximity to humans, amylase gene copy number and amylase activity. Animals that live alongside humans have diets that are different to those of their wild relatives, and these differences have led to dietary adaptations. While wolves (**A**) are highly carnivorous, dogs (**B**) have adapted to eating starchy human food scraps. (**C**) Pajic et al. found that species that consume medium or high amounts of starch have higher amylase activity in their saliva (maroon line) and more amylase gene copies (blue line) than species that consume little or no starch.

Image credit: A. NPS Photo/Ken Conger (CC BY 2.0); B. Mareike Janiak (CC BY 4.0)

Now, in eLife, Stefan Ruhl and Omer Gokcumen of the University at Buffalo and colleagues, including Petar Pajic as first author, report that dogs are not the only species living alongside humans that have adapted to eating more starch (Pajic et al., 2019). The team collected data on amylase gene copy numbers and salivary amylase activity from a range of different mammals, many of which had never been measured before. For several species, both the wild and domesticated (or at least commensal) varieties were sampled, such as wolves and dogs, wild and house (or street) mice and rats, and boars and pigs.

The analyses show that species living in close quarters with humans have more copies of the amylase genes than their wild relatives, as well as higher levels of amylase activity in their saliva. This is even the case for species whose ‘natural’ diets already contained a fair amount of starch, such as mice. Compared to wild deer mice, house mice have even more amylase genes and activity, possibly because of the many starchy foods we (unwittingly) provide.

The results also offer new insights into the ways copy number variations might affect the activity of amylase in saliva. While the number of amylase genes increases almost linearly with starch consumption, there is no difference in amylase activity in the saliva of species with intermediate or high starch consumption. Both have much more amylase in their saliva than species with very low or no starch intake, but in species with high starch consumption, the additional copies of the amylase gene do not seem to translate to higher amounts of salivary enzyme (Figure 1C). Similarly, some species with very high amylase activity in their saliva, such as baboons or macaques, do not have a corresponding increase in amylase gene copies. These unexpected findings should encourage research into the other mechanisms that may affect the activity of salivary amylase. Another avenue of study could be to look at the pancreatic activity of the enzyme. Pajic et al. – who are based in Greece, Germany and several institutes in the US – only measured salivary amylase, but the enzyme is also produced in the pancreas: for example, this is where dogs translate their many amylase gene copies into amylase activity (Axelsson et al., 2013).

Humans have adapted to efficiently digest new foods such as milk and grains (Janiak, 2016), but our dietary habits and our love for starchy carbohydrates might also have shaped the animals that live amongst us. The new findings by Pajic et al. therefore illustrate how powerful our behaviors are at influencing our environment.
